# Rational drug design, medicinal chemistry, planned serendipity and the impact of automation on the drug discovery process

**DOI:** 10.1155/S1463924693000033

**Published:** 1993

**Authors:** Seán O'Connor

**Affiliations:** Bristol-Myers Squibb Pharmaceutical Research Institute Wallingford CT 06292 USA


Journal of Automatic Chemistry, Vol. 15, No. 1 (January–February 1993), pp. 9–12

### Rational drug design, medicinal chemistry, planned serendipity and the impact of automation on the drug discovery process

**Seán O'Connor**
*Bristol-Myers Squibb, Pharmaceutical Research Institute, Wallingford, CT 06292, USA*

This paper examines the processes by which new drug candidates are discovered, especially the early stages of this discovery process. Excluding compounds that are licensed-in, three distinct sources of lead compounds are generally recognized:

(1) Medicinal chemistry.

(2) Empirical screening.

(3) Rational drug design.

The salient features of each approach are first discussed. The evolution of a set of regulatory and business conditions that seemed to favour one of these approaches to the detriment of the others are next examined; finally, the way in which recent developments in automation may have served to establish a new equilibrium between these three lead discovery systems is described.

### Medicinal chemistry

Medicinal chemistry is the first of the modern drug discovery methods. It has given us many of the medicines available today. At its best medicinal chemistry combines a comprehensive knowledge of the medicinal chemistry, synthetic chemistry and pharmacology literature with the ability to imagine what no one else has imagined and then the drive to reduce the concept to synthetic practice. The best of the medicinal chemists also have the ability to think somewhere beyond three dimensions and are able to stretch analogous reasoning way beyond normal limits. A good example of medicinal chemistry is the program that led from Asperlicin (figure 1), a weak, fermentation-derived cholecystokinin antagonist to an orally active compound (figure 2) 16 000 times as potent [1]. The major limitation of medicinal chemistry is that what one starts with is already a known compound. It is therefore inherently less innovative than either of the other two methods discussed in this paper.

### Empirical screening

Empirical screening is also called non-selective, random, blind, mass and broad screening. The object of every real screener, whether he or she is conscious of it or not, is to screen 'all of creation'. 'Numbers' is the name of the game. No one ever accused screeners of being choosey. The unique advantage of this approach is that many of the active compounds discovered are of totally unexpected structures. Good examples are the previously mentioned Asperlicin and the anti-hypercholesterolemic twins, Mevinolin and Pravastatin (see figures 3 and 4).

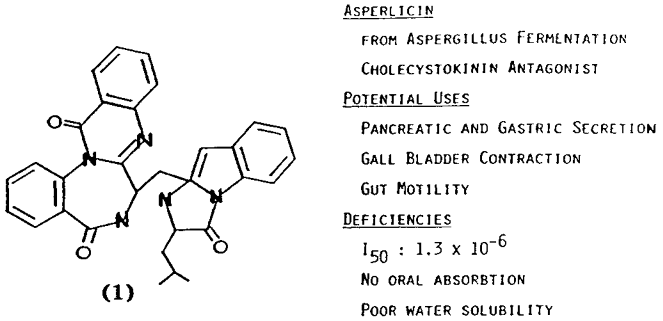

(1)

*Figure 1. Asperlicin.*

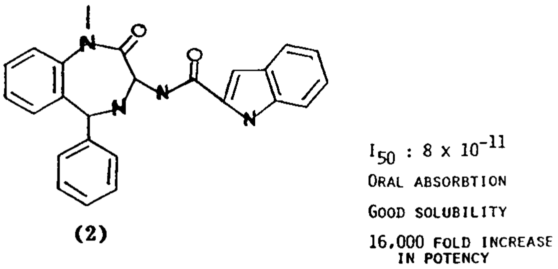

(2)

*Figure 2. Synthetic chemistry based on original structure.*

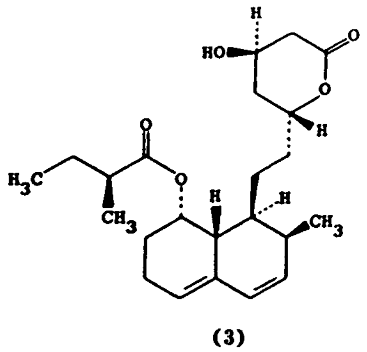

(3)

*Figure 3. Mevinolin.*

This paper was read at the International Symposium on Laboratory Automation and Robotics (October 1992, Boston, USA).

© Zymark Corporation 1993

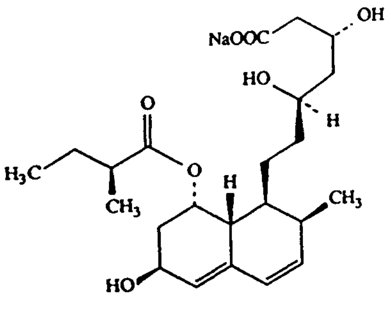

**(4)**

*Figure 4. Pravastatin.*

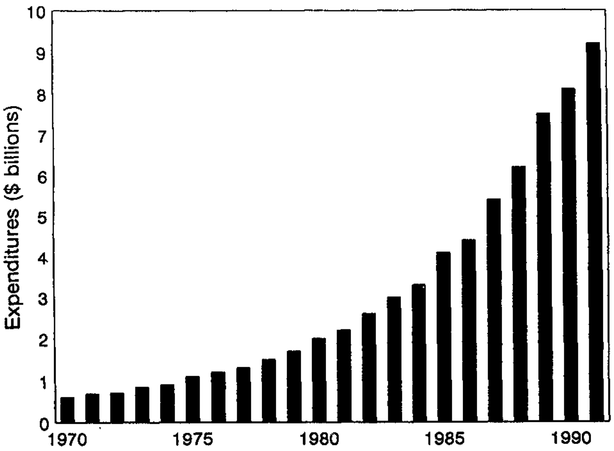

*Figure 5. R&D Expenditures for PMA member firms.*

### Rational drug design

This approach is based on the very reasonable assumption that if one knew the structure of a natural ligand and of its receptor, one should be able to say something useful about the kind of molecules that might mimic or antagonize the activity of the natural ligand. In this approach much reliance is placed on:

(1) X-ray crystallography.
(2) Very high-field NMR.
(3) Computer assisted molecular modelling.
(4) Quantum mechanical calculations.
(5) Cloning and expression of receptors.
(6) Peptidic ligands.

Not all of this is the 'new approach' that many people think it is. By the mid 1960s, Max Marsh at Eli Lilly had already set up a quantum chemistry group that successfully applied quantum mechanical calculations to a real medicinal chemistry SAR. The fact that they needed the corporate mainframe for weekend after weekend to do the analysis on just a few compounds may be one reason this effort never had a sustained impact on the Lilly research programmes. Although today's computers are much more powerful than those of 1965, the time required to carry out a complex calculation is still inordinately long. To model 100 picoseconds of the dynamics of the HIV-1 protease dimer (a total of 198 amino-acid residues), required 90 hours on the Cray computer at Wesleyan University [2].

Rational Drug Design (RDD) programmes have not, as yet, many successes to point to but interesting and potentially useful application have been described for inhibitors of Renin [3], HIV-1 Protease [4] and the Carbonic Anhydrase associated with glaucoma [5].

Several factors have contributed to 'Rational' approach growing from virtually nothing in 1980 to its current significant size:

(1) Rapidly increasing costs.
(2) Longer development times.
(3) The 'mega-drug' phenomenon.

Complicating the situation is what I see as an ironic result of a public relations campaign undertaken by our industry. The ethical pharmaceutical industry has had a long-standing campaign to inform the public of the difficulties inherent in discovering and developing a new drug. This campaign, run by the Pharmaceutical Manufacturer's Association (PMA), has placed great emphasis on the low odds of finding a usable compound. Typically the odds are given as 1 : 10 000, although an Eli Lilly VP (Mitchell Daniels) suggested recently (*Scrip*, April 1992) that the real odds are closer to 1 : 50 000. The public is then told that: 'we have to synthesize/screen 10 000 or even 50 000 compounds in order to discover one drug and then we have to spend 8 years and $200 000 000 to bring it to market'.

Probably the actual numbers are, to some extent, unimportant, but I believe that some, at least, in our industry came to accept the PMA mantra as fact. Faced as they were with rapidly escalating costs, the prospect of a new approach that might reduce costs and shorten development times was very seductive.

Rational Drug Design has been the object of enormous hype. A particularly florid example is the following from a *Forbes* article of 2 April 1991 by Julie Pitta.

'By the end of the decade such "rational drug design" may account for half the research dollars spent at large pharmaceutical houses . . . Apart from producing drugs with fewer unwanted side effects.'

Or the prediction from a corporate VP (Brian Metcalf, SKB, quoted in *Scientific American*, January 1990):

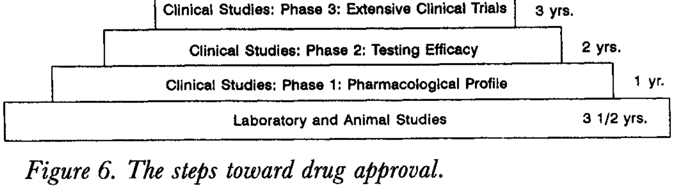

*Figure 6. The steps toward drug approval.*

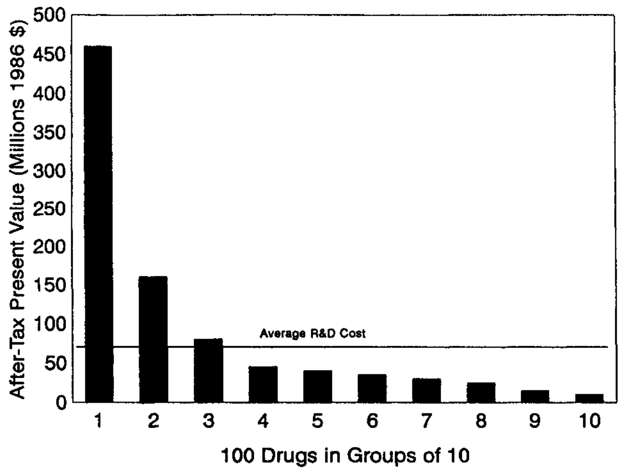

*Figure 7. Earnings performance of 100 drugs versus R&D cost.*

'We see "rational drug design' as the key to the future. We are close to exhausting the traditional approaches.'

No major pharmaceutical company, that I know of, reveals how much of its R&D budget is devoted to the rational approach, although there are several small companies that are so completely dedicated to this approach that one can assign essentially all their research budgets to RDD [6].

In order to get some idea of the magnitude of the investment being made by the major American pharmaceutical companies, I have used information supplied by colleagues at a number of pharmaceutical companies. This was then supplemented by trade information from the CADD hardware and software industry whose sales are significantly affected by the rate of spending by pharmaceutical companies on RDD.

This admittedly hit-and-miss approach yields numbers in the range 5 to 10% of the drug discovery budget being spent on RDD approaches in 1991. With a number of R&D budgets slated to reach or exceed the $1 billion mark in 1993 and assuming 20–25% being spent on 'R', one can calculate that $10 to $25 M a year is being spent on Rational Approaches for every $1 billion in R&D expenditure. The member firms of the PMA will spend $10 billion on R&D in 1993. This translates into something in excess of $100 M for Rational Drug Design programmes.

Between 1980 and 1990, the US pharmaceutical industry R&D budget grew at an average annual rate of 15%. From 1984 to 1990, rational drug design budgets appear to have grown at over 20% a year. There are indications, however, that this rate has slowed in the past two years. Few believe that Pitta's prediction of only last year will be fulfilled. So, what, if anything, has happened?

Apart from the relative lack of measurable success from the RDD approach, a failure that has been unfortunately magnified by the hype I referred to earlier, something else was happening during the 1980s. Some of us in the industry believe that its impact will be at least as significant as that of RDD. I am referring, of course, to laboratory automation with particular emphasis on material handling equipment.

When I departed Eli Lilly in 1982 they were still running their fermentation screens in agar. At that time both Lilly and Bristol-Myers (Tokyo) were running 100 to 200 fermentations a week, i.e. 5000 to 10 000 fermentation samples a year in, perhaps, 10 assays (screens) and a clear distinction was made between fermentation samples and synthetic compounds from the corporate files. Between 1980 and 1990 the whole world of screening changed as reflected by William Netzer in an article in the July 1990 *Biotechnology*, in which he describes Merck as 'perhaps the most prolific screener' claiming that they are now screening' as many as 40 000 samples a year in as many as 60 screens'. But Merck is by no means alone in this. Eli Lilly now has a well-equipped system distributing samples, both natural and synthetic, for screening. Similar systems already exist, or are being developed, in most major pharmaceutical companies, including Bristol-Myers Squibb. Overall, the sample throughput capability appears to have increased 5 to 50 fold since the 'old days' of a decade ago. During the same period the manpower required for the running of a typical screen has probably decreased slightly. The question for us here today is 'how did this come about?'

There were a number of important contributors to this amazing increase in productivity. The single most important factor, in my opinion, was the development and widespread use of the 96-well microplate. In terms of importance this is closely followed by the introduction of multi-channel pipettes, followed by the first of the programmable pipetters, the serial diluters, the microplate readers, mini-barcode printers and readers, programmable material handling systems and the use of robots in a fetch-and-carry mode to permit overnight operation.

The result of all of this is that even if the gloomy statistics of 1 in 50 000 were correct, by using a combination of fermentation broth-derived samples and synthetic compounds from corporate archives or other sources, one should be able to find one to two new leads every year in every screen.

At the beginning of this paper, the establishment of a new equilibrium between the three major lead discovery approaches was mentioned. In several companies, this seems to have already occurred, due largely to the revolution in screening automation and the steady stream of novel leads that have emerged from the screening programmes.

A good example of this is a recent target that was attacked simultaneously by each of the three approaches.

Endothelin, the most potent endogenous pressor agent (blood-pressure raising compound) was first reported by Yanagisawa in 1988. It is a 21-AA peptide whose sequence and synthesis were also reported that same year. Many medicinal chemistry groups immediately started peptide synthesis programmes to delineate the requirements for binding to the receptor and for functional activity. The results of this approach have not been particularly useful or revealing since, almost by definition, peptides do not have many of the properties desired in a drug.

Meanwhile, the rational designers set out to clone one or more of the ET receptors. This was not accomplished until 1992. The receptor has still not been crystallized and, therefore, no X-ray structure data is yet available.

While all this was going on, Merck-Banyu, Fujisawa, Bristol-Myers Squibb and an unknown number of other companies were screening everything in sight for compounds that would specifically inhibit the binding of endothelin to its receptor.

In May 1991 a note from Banyu (Merck) in Tokyo reported the isolation and characterization of a fermentation-derived antagonist [7]: It is a cyclic pentapeptide whose structure quickly became the focus of a successful medicinal chemistry programme. A rough guess would be that Banyu discovered their antagonist in January 1990. Banyu's report was followed by one from Fujisawa [8]. They had found the same cyclic peptides and subsequently found a non-peptide antagonist [9]. I expect by now everyone has some kind of a lead from either fermentation or synthetic screening or, as at Bristol-Myers Squibb, from both.

The extraordinary sample handling and screening capacities that are now integral to the research programmes of a number of the major pharmaceutical companies have changed the world of lead discovery. We now know with a fair degree of certainty that, given a year, we can supply a novel lead for almost any target or research programme. We cannot guarantee that the lead will necesssarily be useful but, then, no one else can make that promise either. The mere possession of such a lead provides a starting point for a medicinal chemistry programme. Rational drug design also benefits from the discovery of these leads, although somewhat less directly. True synergy between the different approaches has been restored and we are all enriched by it.

Today's problems are no longer concerned with throughput, instead we now find ourselves running out of materials to screen. Fortunately, soil micro-organisms are probably generating new strains faster than we can screen them and, given the need for fresh sources of screening samples, what better time than now for the rain forests to come into their own as unique sources of secondary metabolites?

### References

1. Goetz, M. A., *et al.*, *Journal of Antibiotics*, **38** (1985).
2. Mansuri, M. M., personal communication (1992).
3. Reily, M. D. *et al.*, *FEBS Letters*, **302** (1992), 1.
4. DesJarlais, R. L. and Craik, C. S. *et al.*, *Proceedings of the National Academy of Sciences*, **87** (1990).
5. Graham, S. L. *et al.*, *Journal of Medicinal Chemistry*, **32** (1989).
6. Companies believed to be wholly or largely dedicated to the 'rational' approach include Vertex Pharmaceuticals, Cambridge, Massachusetts, Agouron Pharmaceutical, La Jolla, California and Arris Pharmaceuticals, South San Francisco, California.
7. Ihara, M. *et al.*, *Biochemistry and Biophysics Research Communications*, **178** (1992).
8. Miyata, S. *et al.*, *Journal of Antibiotics*, **45** (1992).
9. Miyata, S. *et al.*, *Journal of Antibiotics*, **45** (1992).

